# Genomic diversity and structure of a Neotropical microendemic fig tree

**DOI:** 10.1002/ece3.11178

**Published:** 2024-03-18

**Authors:** Ángela P. Rojas‐Cortés, Jaime Gasca‐Pineda, Antonio González‐Rodríguez, Guillermo Ibarra‐Manríquez

**Affiliations:** ^1^ Instituto de Investigaciones en Ecosistemas y Sustentabilidad Universidad Nacional Autónoma de México Morelia Michoacán Mexico; ^2^ Posgrado en Ciencias Biológicas Universidad Nacional Autónoma de México, Ciudad Universitaria Ciudad de México Mexico; ^3^ Departamento de Ecología Evolutiva Instituto de Ecología, Universidad Nacional Autónoma de México, Circuito exterior s/n anexo al Jardín Botánico, Ciudad Universitaria Ciudad de México Mexico

**Keywords:** *Ficus*, gene flow, Mexican Transition Zone, population structure, tropical dry forest

## Abstract

Genetic diversity is a key component of evolution, and unraveling factors that promote genetic differentiation in space and time is a central question in evolutionary biology. One of the most diverse and ecologically important tree genera in tropical forests worldwide is *Ficus* (Moraceae). It has been suggested that, given the great dispersal capacity of pollinating fig wasps (Chalcidoidea; Agaonidae), the spatial genetic structure, particularly in monoecious fig species, should be weak. However, no studies have addressed the factors that determine the genetic structure of *Ficus* species in regions of high geological, geographic, and climatic complexity, such as the Mexican Transition Zone. Using nuclear single nucleotide polymorphisms (5311 SNPs) derived from low‐coverage whole genomes and 17 populations, we analyzed the population genomics of *Ficus pringlei* to characterize neutral and adaptive genetic variation and structure and its association with geographic barriers such as the Trans‐Mexican Volcanic Belt, environmental heterogeneity, and wind connectivity. From genomic data of 71 individuals, high genetic diversity, and the identification of three genomic lineages were recorded (North, South, and Churumuco). The results suggest that genetic variation is primarily determined by climatic heterogeneity. *Ficus pringlei* populations from the north and south of the Trans‐Mexican Volcanic Belt also exhibited minimal genetic differentiation (*F*_ST_ = 0.021), indicating that this mountain range may not act as an insurmountable barrier to gene flow. Wind connectivity is also highlighted in structuring putative adaptive genetic variation, underscoring the intricate complexity of the various factors influencing genetic variation in the species. This study provides information on the possible mechanisms underlying the genetic variation of endemic species of the tropical dry forest of Western Mexico, such as *F*. *pringlei*.

## Graphical abstract

The population genomics of *Ficus pringlei* (Moraceae) is analyzed to characterize neutral and adaptive genetic variation and structure and its association with geographic barriers and environmental heterogeneity. This study provides information on the diversity and the mechanisms underlying the genomic variation of *F. pringlei*. Although a weak or negligible genetic structure is often assumed for the *Ficus* species, in *F. pringlei* we detected three distinct genomic lineages, suggesting limited gene flow influenced mainly by climatic conditions.

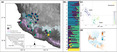

## 1 INTRODUCTION

Genetic diversity is one of the essential components of biological diversity, and unraveling the factors that contribute to its origin and maintenance is a central question in evolutionary biology (Amos, 1998). Levels and geographic distribution of genetic diversity can be shaped by factors such as spatial isolation, climatic heterogeneity, and geographic barriers. However, characteristics of the life history of the species can also lead to population divergence (Gelmi‐Candusso et al., 2017; Hamrick et al., 1992). It has been suggested that widely distributed, wind‐pollinated woody species have greater diversity and less differentiation between populations than endemic, animal‐pollinated species with other life forms (Dick et al., 2007; Hamrick et al., 1992). Therefore, the study of genetic diversity and the factors that shape it allow a better understanding of the complex evolutionary histories of species.

*Ficus* L. (Moraceae) is a genus with high species diversity and great variation in ecological characteristics that can shape genetic diversity patterns (Beech et al., 2017; Heer et al., 2015; Nazareno et al., 2013). Each of the approximately 800 *Ficus* species has an obligate pollination system carried out exclusively by wasps of the Agaonidae family, which can only reproduce in the pistillate flowers of the fig inflorescence or syconium (Janzen, 1979; POWO, 2023; Ramírez, 1970). These wasps are generally highly mobile dispersers that rely on passive dispersal by wind. However, this mobility is not only dictated by wind but is also affected by factors such as the sexual system of the plants (Ahmed et al., 2009; Nason et al., 1998; Nazareno et al., 2013).

Wasps associated with monoecious trees disperse farther than those of dioecious species due to differences in tree height and clustering. Monoecious *Ficus* are generally tall and scattered, while dioecious *Ficus* are smaller and spatially aggregated. Thereby, monoecious *Ficus* species have generally been associated with weak genetic structures due to high gene flow, limited adaptation to local environments, and little or no evidence of isolation by distance between populations (Bain et al., 2016; Nazareno et al., 2013; Yang et al., 2015). However, despite their wide distribution and high dispersal capacity, some Neotropical trees can show significant genetic differentiation, often influenced by strong geographic barriers such as mountains or rivers, which promote isolation by distance (Heer et al., 2015; Honorio Coronado et al., 2014; Lowe et al., 2018). Furthermore, not all monoecious species have extensive distributions (e.g., *F*. *blepharophylla* Vázq. Ávila, *F*. *lapathifolia* (Liebm.) Miq. or *F*. *petiolaris* Kunth; Ibarra‐Manríquez et al., 2012; Pederneiras et al., 2023). Those species with localized distributions could present limited genetic dispersion, generating a more marked genetic structure, signs of local adaptation, and even a more notable influence of genetic drift (Loveless & Hamrick, 1984), in contrast to widely distributed monoecious species.

The Neotropics encompass many biomes with a particular history of landscapes and biotic evolution (Hughes et al., 2013). The Mexican Transition Zone, where the Nearctic and Neotropical regions converge (Anguiano‐Constante et al., 2021; Halffter & Morrone, 2017), is characterized by great geological, geographic, and climatic complexity, and has contributed to the genetic variation of species and is considered as a megadiversity region. In this area, it has been suggested that recent mountainous complexes, such as the Trans‐Mexican Volcanic Belt (TMVB), are main drivers of diversification in Mexico and have affected taxa in different ways (Mastretta‐Yanes et al., 2015). For example, in the lowlands, the TMBV has functioned as a barrier by introducing a genetic discontinuity in several species with different dispersal capabilities (Arbeláez‐Cortés et al., 2014; López‐Barrera et al., 2021; Schramm et al., 2021; Zarza et al., 2008). However, the pattern of genetic diversity and structure in the *Ficus* species of this region is unknown.

Within the Mexican Transition Zone is *Ficus pringlei* S. Watson (subg. *Spherosuke* Raf., sect. Americanae (Miq.) Corner), a monoecious Mexican endemic species (Ibarra‐Manríquez et al., 2012). This species primarily occurs in the dry tropical forests of Western Mexico, characterized by high biological diversity and deforestation rates (Ceballos et al., 2010; Ceballos & Garcia, 1995; Rojas‐Cortés et al., 2022). The distribution of this species is fragmented by the highlands to the west of the TMVB and the northeast of the Sierra Madre del Sur (Rojas‐Cortés et al., 2022). In addition to these geographical features that can act as barriers, in the northern areas of the TMVB, a higher degree of seasonality of temperature has been observed (Rojas‐Cortés et al., 2022), which could contribute to the differentiation and heterogeneity within the distribution of the species. Nonetheless, the genetic diversity of this species and how mountain systems and environmental heterogeneity can shape it is yet to be determined.

This is the first study to investigate the population genomics of a Neotropical *Ficus* species using single nucleotide polymorphisms (SNPs). Our objectives are twofold: (a) to characterize neutral and adaptive genetic variations and structures within *F. pringlei* and (b) to assess the impact of environmental, geographic, and wind‐related factors on genetic variation patterns. We hypothesize that geographic isolation, enforced by physical barriers like TMVB and Sierra Madre del Sur, diminishes gene flow, leading to the differentiation between populations north and south of these barriers. We also anticipate a correlation between climatic variations and limitations on the dispersal of pollinators or seed dispersers with patterns of environmental variation and genetic structure. Considering the significant role of wind in pollinator dispersal, we also hypothesize that specific wind patterns and directions play a crucial role in shaping the genetic structure of populations.

## 2 METHODS

### 2.1 Study system

*Ficus pringlei* distributes in Colima, Guerrero, Jalisco, Michoacán, Nayarit, and Zacatecas states, in the Pacific coast and Central Mexico (Rojas‐Cortés et al., 2022). Unlike most species of the subgenus *Spherosuke*, which have a hemiepiphytic or strangler habit, *F*. *pringlei* is rupiculous, growing on rocky outcrops or cliffs, reaching up to 12 m in height (Ibarra‐Manríquez et al., 2012). The figs develop in the axil of the leaves and, when ripe, are red, ca. 1.5 cm in diameter and suggesting mainly bird dispersal (Lomáscolo et al., 2008; Rojas‐Cortés et al., 2022). As in other *Ficus* species, the development of figs in *F*. *pringlei* is coupled with the wasp's life cycle (Rojas‐Cortés et al., 2022). In phase B or female, wasps of the genus *Pegoscapus* Cameron 1906 oviposit and pollinate flowers with receptive stigmas. In phase D or male, the new generation of female wasps leaves the syconium laden with pollen. The development of figs within the crown of the trees of this species can be asynchronous, so there are in a given tree syconia in different stages of development (Rojas‐Cortés et al., 2022), or synchronous.

### 2.2 Sampling, DNA extraction, and sequencing

**FIGURE 1:**
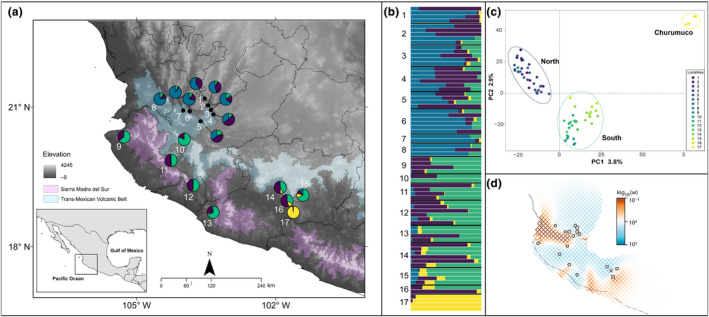
Geographical population structure and migration surface for *Ficus pringlei*. (a) Geographic distribution of 17 sampling localities. For more detailed information about sampling locations, see Table 1. Circles represent the assignment proportion to the four genomic lineages identified in sNMF. (b) Individual ancestry coefficient for 71 individuals obtained with sNMF, *K* = 4, and based on 5311 SNPs; each vertical line depicts one individual. (c) Structure of *F*. *pringlei* using principal component analysis (PCA), with each point colored according to its respective locality. Each dot indicates one individual and is colored according to sampling localities. (d) Estimated effective migration surface plots. Highlighted blue regions have higher‐than‐expected migration rates, and orange‐shaded regions have lower‐than‐expected migration rates. Points represent sampled locations.

Through the distribution of *F*. *pringlei*, the young leaves of 71 individuals were sampled from 17 localities between 2018 and 2020 (Table 1, Figure 1a). In each plant, between 3 and 4 young leaves were dried in silica gel and voucher specimen was collected and deposited in the National Herbarium of Mexico (MEXU) and the Herbarium of the Missouri Botanical Garden (MO).

**TABLE 1:** Sampling site information and genetic estimates at the locality and genomic lineage level based on 5311 SNPs.

Localities	Longitude	Latitude	State	*N*	*H*_e_ (SD)	*H*_o_ (SD)	*F*_IS_
North lineage				35	0.239 (0.131)	0.264 (0.168)	−0.088
1. Zacatecas	−103.5322	21.1791	Zacatecas	4	0.191 (0.186)	0.237 (0.270)	−0.059
2. San Cristóbal	−103.4583	21.0313	Jalisco	5	0.218 (0.173)	0.265 (0.270)	−0.084
3. Huaxtla	−103.3959	20.9374	Jalisco	5	0.223 (0.172)	0.281 (0.256)	−0.122
4. Guadalajara	−103.3432	20.8383	Jalisco	6	0.210 (0.166)	0.238 (0.224)	−0.024
5. La Primavera	−103.6193	20.6925	Jalisco	4	0.191 (0.185)	0.239 (0.267)	−0.070
6. Tequila	−103.8515	20.9050	Jalisco	6	0.228 (0.167)	0.283 (0.246)	−0.128
7. Magdalena	−103.9955	20.9225	Jalisco	2	0.197 (0.212)	0.300 (0.362)	−0.123
8. Nayarit	−104.4834	21.1513	Nayarit	3	0.210 (0.195)	0.293 (0.315)	−0.145
South lineage				32	0.236 (0.131)	0.258 (0.165)	−0.071
9. El Tuito	−105.3298	20.3814	Jalisco	4	0.207 (0.185)	0.269 (0.279)	−0.125
10. Colotepec	−103.9676	20.2403	Jalisco	2	0.164 (0.213)	0.272 (0.382)	−0.217
11. Autlán	−104.2520	19.8332	Jalisco	4	0.198 (0.182)	0.244 (0.265)	−0.062
12. Colima	−103.7578	19.2604	Colima	6	0.218 (0.167)	0.259 (0.233)	−0.081
13. Coalcomán	−103.3465	18.7177	Michoacán	4	0.217 (0.182)	0.280 (0.274)	−0.117
14. Cuatro Caminos	−101.9175	19.2187	Michoacán	6	0.206 (0.170)	0.233 (0.225)	−0.021
15. Chipícuaro	−101.4025	19.1363	Michoacán	4	0.220 (0.184)	0.291 (0.283)	−0.143
16. La Huacana	−101.7515	18.9725	Michoacán	2	0.136 (0.203)	0.203 (0.342)	−0.027
17. Churumuco	−101.6605	18.7015	Michoacán	4	0.181 (0.203)	0.277 (0.347)	−0.328

Abbreviations: *F*_IS_, Inbreeding coefficient; *H*_e_ (SD), Expected heterozygosity (standard deviations); *H*_o_ (SD), observed heterozygosity (standard deviations); *N*, number of individuals.

Genomic DNA was isolated from 50 mg of dry leaf tissue using a standard protocol (Doyle & Doyle, 1990). Prior to the DNA extraction process, a prewash step was implemented using a washing buffer containing Tris–HCl, EDTA, NaCl, 2‐mercaptoethanol, and polyvinylpyrrolidone, aiming to eliminate secondary compounds (Li et al., 2007). DNA concentrations were quantified with a Qubit (Thermo Fisher Scientific, MA, USA) and the quality of the extraction with NanoDrop 2000 (Thermo Fisher Scientific). DNA was diluted with TE buffer to obtain approximately 10 ng/μL. The extractions were sent to SNPsaurus LLC (Eugene, OR) for library preparation and low‐coverage whole‐genome sequencing.

A complete genome library was prepared with 5 ng of genomic DNA, fragmented with the Nextera DNA Flex reagent (Illumina, Inc), which also ligates the adapter sequences to the ends of the fragments. Whole genome sequencing was made to an average of 8× read depth on a NovaSeq 6000 S4 lane (Illumina, Inc., San Diego, CA, USA), with 150 bp reads from paired ends.

### 2.3 Bioinformatics and genotyping

First, reads from each sample were trimmed using the BBDuk of BBTools (Bushnell, 2020). Trimming removed the Nextera adapter sequences, and quality‐trimmed the end of the reads as needed. Next, the reads were extended using tadpole (BBTools) with default parameters, and the extended reads were used as input for the *novo* assembly of the sample with more raw reads (29,272,027) in SPAdes (Prjibelski et al., 2020) with a *k* = 104. For each individual, the reads from the organelles were removed using NOVOPlasty (Dierckxsens et al., 2016).

Alignments in SAM format were sorted, indexed, and compressed in bam format using SAMTOOLS version 1.9 (Danecek et al., 2021) and nest processed with Picardtools (Broad Institute, 2019). The Picard processing validated read pairing, removed duplicate reads, and added read groups for analysis in the Genome Analysis Toolkit (GATK, Poplin et al., 2018). The call of genotypes was performed in GATK Unified Genotyper v.4.2 using as assembly reference the contigs with 10,000 base pairs or more and the best practices workflow by individuals VCFs using ‘HaplotypeCaller’, specifying–ERC GVCF, and then calling population variants using ‘GenotypeGVCFs’ (Van der Auwera et al., 2013). We then used GATK ‘VariantFiltration’ to further hard filter based on GATK's best practices recommendation (QD < 2.5, QUAL < 40.0, SOR > 2.5, FS > 50.0, MQ < 50.0, MQRankSum < −10.5, ReadPosRankSum < −4.0), and used GATK ‘SelectVariants’ to include only variants that met all filtering thresholds. Sets of variants were further filtered using vcftools v.0.1.15 (Danecek et al., 2011) to remove SNPs with low‐frequency alleles in the population (MAF < 0.05), with more than two alleles at a single position, or in linkage disequilibrium (window size 100, step size 100 and *R* ^2^ threshold .25). Also, we filtered out SNPs outside Hardy–Weinberg Equilibrium (*p*‐value .001), with mean depth values lower than 10, with a proportion >0.75 of missing data, and a final step was taken to reduce paralogous sections of single SNPs by applying a threshold of 250 base pairs (thin 250).

### 2.4 Diversity and population structure analyses

The calculation of observed (*H*_o_) and expected (*H*_e_) heterozygosity, along with the coefficient of inbreeding (*F*_IS_), was performed for each locality and the genomic lineages identified by principal component analysis (PCA). The dartR package (Gruber et al., 2018) was employed for these estimations. Pairwise relatedness was calculated using vcftools for all individuals, where a negative value indicates genetic dissimilarity, a value close to 0 indicates no significant or apparent genetic relationship, and a value close to 0.5 indicates high genetic similarity (Manichaikul et al., 2010).

Population structure was assessed using PCA on all individuals using the adegenet package (Jombart, 2008). The individual ancestry coefficients were estimated based on sparse non‐negative matrix factorization algorithms (sNMF) through the package LEA (Frichot & François, 2015). The sNMF is comparable to other widely used programs, such as Admixture and Structure but is computationally faster and robust to departures from traditional population genetic model assumptions, such as Hardy–Weinberg equilibrium (Frichot & François, 2015). Ancestry coefficients were estimated for 1–15 ancestral populations (*K*) using 200 replicates for each *K*. The cross‐entropy criterion was then used to determine the best K based on the prediction of masked genotypes.

Overall F_ST_ was calculated in the hierfstat package (Goudet, 2004). Pairwise genetic distance among localities and genetic distance between genomic lineages suggested by PCA were estimated by Weir and Cockerham's *F*_ST_ (Weir & Cockerham, 1984) using StAMPP (Pembleton et al., 2013). All analyses were performed in R v 4.2.2 (R‐Core‐Team, 2022). The spatial method Fast and Flexible Estimation Effective Migration Surfaces (FEEMS, Marcus et al., 2021) was implemented to detect patterns of genetic diversity across a landscape that deviates from a null expectation of isolation by distance (IBD). This method was applied as an exploratory tool to find regions of the landscape that may act as biogeographic barriers in this system (e.g., The TMVB or/and Sierra Madre del Sur).

### 2.5 Environmental data

Nineteen environmental variables were initially used to investigate the relationship between genetic variation and environmental gradients. These variables were obtained from high‐resolution monthly climate surfaces in geographical coordinates (Datum WGS‐84), with a spatial resolution of 3 arc seconds (∼90 m) for the 1910–2009 period (Cuervo‐Robayo et al., 2014). A correlation test with a threshold of 0.85 was used to mitigate the problem of collinearity among the variables. This implies that if a strong correlation was found between two variables, only the one with the highest representativeness of climatic conditions throughout the year and influence on the seasonal environment of *F*. *pringlei* was retained.

### 2.6 Identification of variation under selection

To enhance the robustness of outlier detection in this study, three methods to detect SNPs under selection were used: Redundancy Analysis (RDA, Capblancq & Forester, 2021; Forester et al., 2018), the latent factor mixed model (LFMM, Caye et al., 2019), and PCAdapt (Luu et al., 2017). RDA has been used as a Genome‐Environment Association (GEA) method with high detection power and a low false positive rate in identifying adaptation signatures (Capblancq & Forester, 2021; Forester et al., 2018). Simple RDA was performed, treating 5311 SNPs as response variables and six environmental variables as explanatory variables. Data were imputed based on sNMF‐estimated ancestry coefficients, using the imputation function of the LEA package to reduce the potential effects of missing sites. Outliers were identified on each of the first three ordination axes as SNPs with a locus score of ±3 SD from the mean score for that axis RDA, as Forester et al. (2018) suggested.

Besides RDA, LFMM, a univariate GEA method, was performed using the R package lfmm (Caye et al., 2019). The number of latent factors was chosen following sNMF‐estimated ancestry coefficients (*K* = 4). As a complement to GEA methods, PCAdapt analysis was applied to identify SNPs putatively under selection pressure because they deviate from the typical distribution of the test statistic *Z* (Luu et al., 2017). Two principal components (*K* = 2) were used for estimating the test statistics. The list of putative adaptive SNPs was obtained for both methods under an expected false discovery rate of *α* = .05 using the R package qvalue (Storey et al., 2022).

A VennDiagram package (Chen & Boutros, 2011) was used to identify all putative adaptive SNPs identified and shared among the three methods of identification of outlier SNPs. Two datasets were built after identifying putative SNPs under selection: neutral and putatively adaptive. The first dataset included all SNPs (5289 SNPs), except SNPs identified by at least two methods of identifying SNPs under selection and the second dataset included 22 SNPs.

### 2.7 Partitioning genomic variation

Redundancy analysis (RDA) was applied independently to neutral and putatively adaptive SNP datasets to evaluate the impact of environmental and geographic variables, and wind connectivity, on genomic variation. Likewise, this analysis encompassed the entire dataset and each genomic lineage identified by PCA, excluding Churumucho due to its limited sample size. The genotypic matrix per individual served as the response variable. To mitigate overfitting, we conducted separate forward selections on environmental and geographic variables using the ordiR2step function. Following this, variance partitioning in RDA was executed using the varpart function. Significance testing for partitioning in all RDA models involved 999 permutations with the anova.cca function. These procedures were implemented using the ‘vegan’ R package (Oksanen et al., 2022).

The initial set of environmental variables, comprising six uncorrelated factors (bio01, annual mean temperature; bio04, temperature seasonality; bio12, annual precipitation; bio17, precipitation of driest quarter; bio18, precipitation of warmest quarter; bio19, precipitation of coldest quarter), was chosen as explained in the environmental data section. Additionally, geographic variables were derived by transforming each individual's longitude and latitude coordinates into distance‐based Moran eigenvector maps (MEM). These MEMs typically describe broad spatial structures, capturing spatial variation across the entire sampled area. Conversely, the last eigenvectors depict fine spatial structures, potentially capturing variation at the scale of sampling sites (Dray et al., 2012). The truncation threshold was set to the length of the longest edge of the minimum spanning tree to compute the MEMs, and only positive values were retained using the adespatial package (Dray et al., 2022).

**FIGURE A1:**
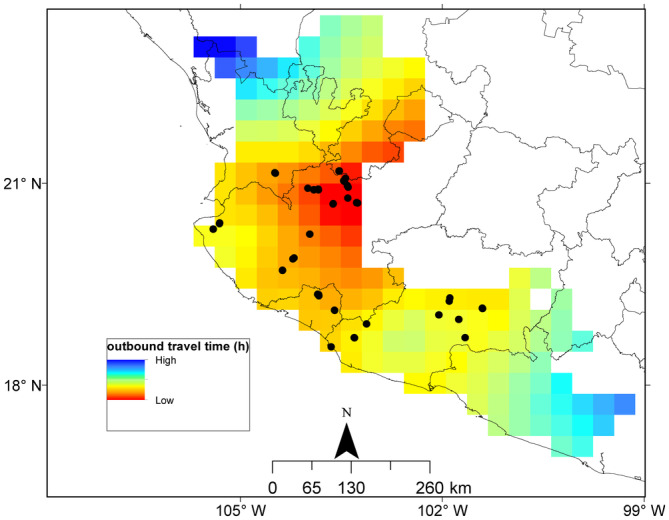
Wind dispersal patterns are represented by one‐way travel time (h) for the area where *Ficus pringlei* is located. Black dots indicate individuals sampled for this study. Warmer colors represent shorter travel times, indicating greater connectivity, while colder colors indicate less connected areas.

Wind connectivity values between individuals were calculated using the R windscape package (Kling, 2023) and three decades (1980–2009) of global hourly wind data from the Climate Forecast System Reanalysis (Saha et al., 2014). For each individual, an accessibility surface illustrating the ease of inbound or outbound wind dispersal was generated. These surfaces depict the estimated average time, in hours, for wind to reach specific locations, considering the spatio‐temporal distribution of wind conditions across the landscape. Subsequently, the values of each connectivity raster per individual were cumulatively summed, resulting in a surface providing an estimated connectivity value for the entire species range. Finally, only individual connectivity values from the outbound surface were extracted for integration into the RDA analyses (Figure A1 in Appendix), considering the reciprocity in wind flow between both directions.

## 3 RESULTS

### 3.1 Genomic diversity and structure analysis

For 71 individuals, 1,171,156,667 reads, and 287 GB of raw data were generated, with an average of 15,615,422.23 ± 5,633,235.98 reads per individual. After filtering, 5311 SNPs were retained. The overall *H*_o_, *H*_e_, and *F*_IS_ values were 0.263, 0.234, and −0.124, respectively. The observed heterozygosity ranged from 0.203 in locality 16 to 0.300 in locality 7, while the average gene diversity (*H*_e_) fluctuated from 0.136 in locality 16 to 0.228 in locality 6. Across all localities and genomic lineages (see below), *H*_o_ consistently exceeded *H*_e_, with negative *F*_IS_ values, indicating an excess of heterozygotes (Table 1).

**FIGURE 2:**
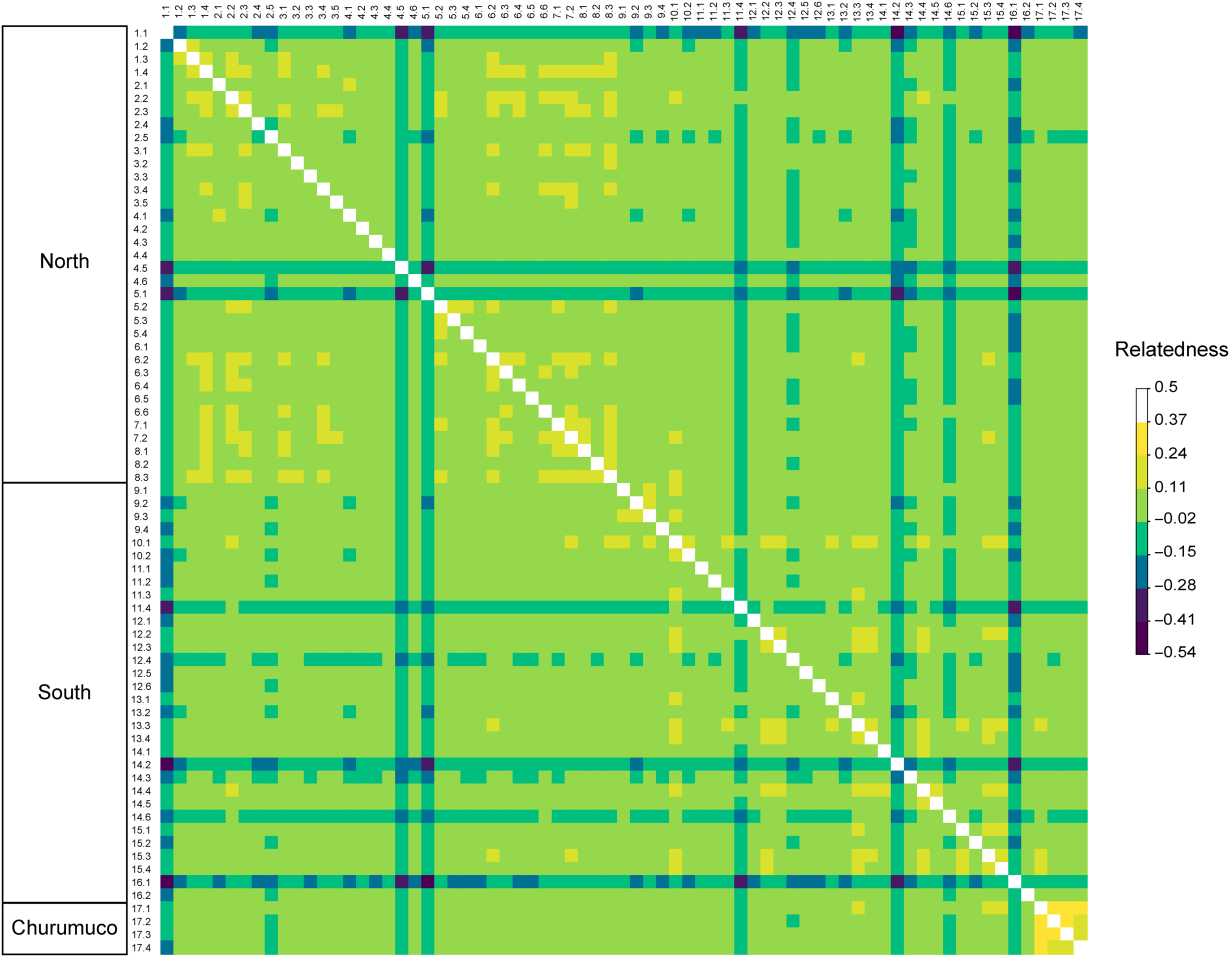
The overview diagram of genomic relatedness between the 71 sequenced individuals.

Relatedness with values >0.26 was found between all individuals from locality 17 (mean 0.27), meaning that these individuals could be parent‐offspring or full sibs. In contrast, 66% of the plants north of the TMVB (relatedness values range −0.35 and 0.21, mean 0.06) and 50% of the plants south of the TMVB (relatedness values range −0.38 and 0.24, mean 0.04) showed at least one value higher than 0.11 with another individual, meaning that could be second‐degree relatives. In addition, seven plants from the north and south of the TMVB (1.1, 4.5, 5.1, 11.4, 12.4, 12.4, 12.4, 14.2, 14.3, 14.6, 16.1) had relatedness values of less than zero, meaning that they are not closely related to other individuals (Figure 2).

**FIGURE 3:**
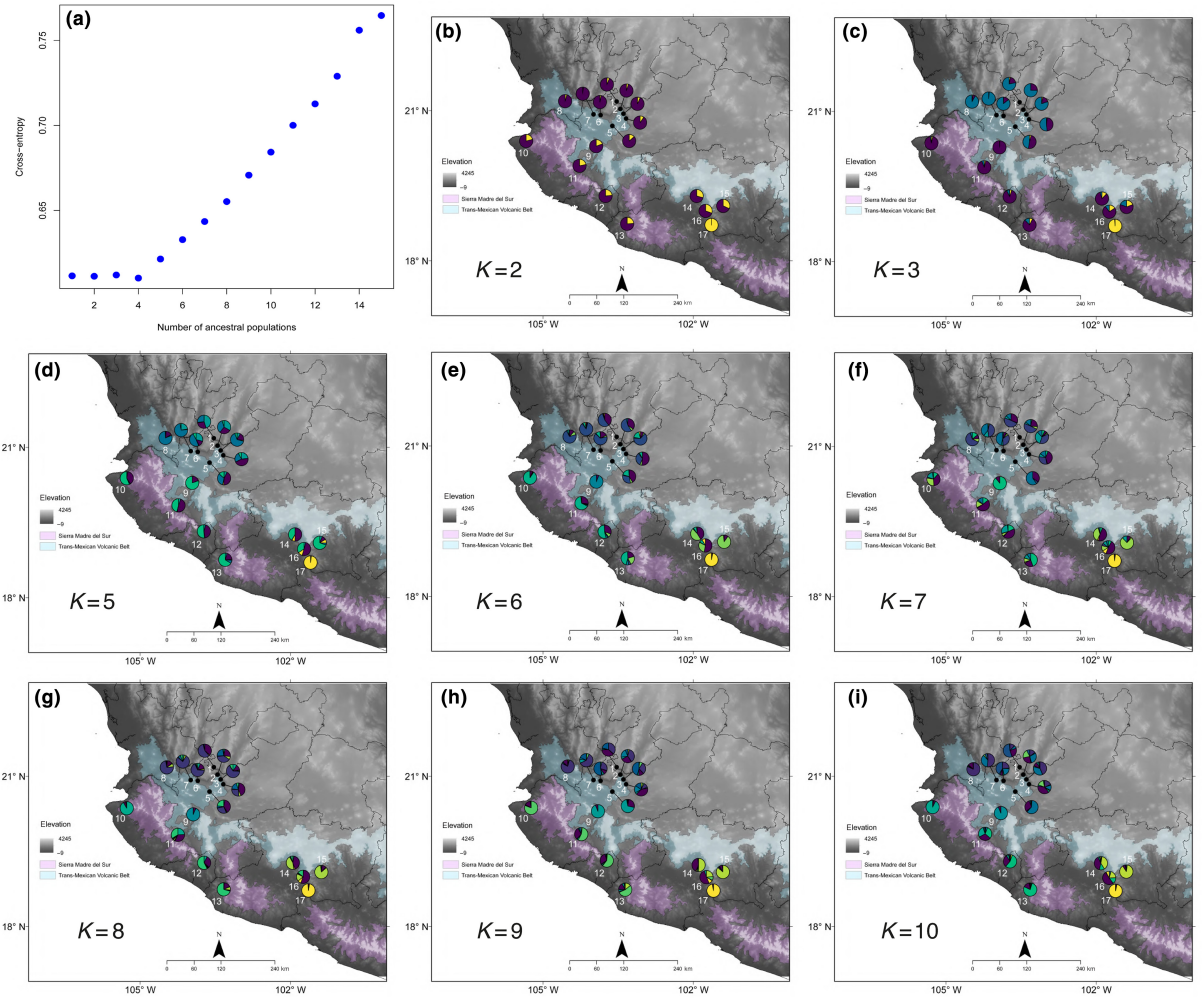
Cross‐entropy and geographical population structure with alternative levels of *K* = 2–10 estimated with sNMF. The lowest value represents the most likely number of genomic lineages in the cross‐entropy. Note that the result for *K* = 4 can be found in Figure 1a.

The first two component axes in the PCA explained 6.7% of the variation depicting three genomic lineages (Figure 1c). The first lineage includes localities North of the TMVB (1–9). Another lineage was formed by sites South of the TMVB (localities 10–16). The third lineage included plants from locality 17 (Churumuco). The sNMF analysis, based on the optimal value *K* = 4 of the cross‐entropy criterion (Figure 3), identified the same three genomic lineages of PCA, in addition to another lineage including plants from all localities (Figure 1a,b).

**FIGURE 4:**
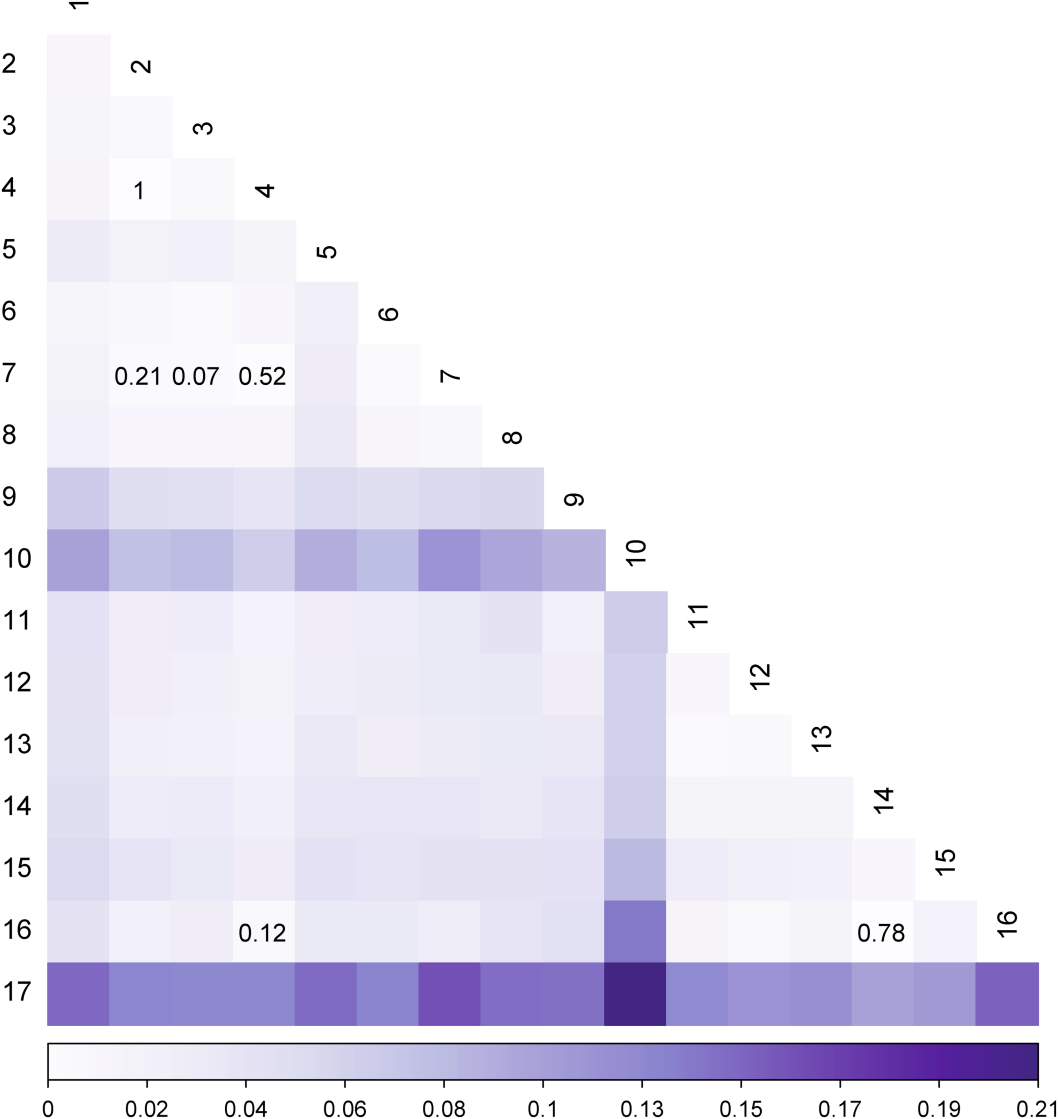
Genetic distances among sampling localities. The numbers in each cell indicate non‐significant *F*_ST_ pairwise values (*p*‐values > .05).

The overall *F*_ST_ value between localities was low (0.042). For most pairs of localities, *F*_ST_ was significant (*p* < .05), with values between 0 and 0.208 (Figure 4). Locality 17 (Churumuco) was less related to other localities (paired F_ST_ ranging from 0.098 to 0.208). The paired F_ST_ between the genomic lineages suggested by PCA (i.e., North, South, and Churumuco) was significant (*p* < .05), with values between 0.021 and 0.099. The highest genetic differentiation was found between the locality 17 with the North (*F*_ST_ = 0.099) and the South (*F*_ST_ = 0.080), while between the North and the South, there was the least differentiation (F_ST_ = 0.021). FEEMS analysis suggested uneven gene flow across the landscape (Figure 1d). Areas with low rates of gene flow corresponded to the uplands to the west of TMVB and Sierra Madre del Sur. Low rates of gene flow were also recorded near locality 12 (Colima) and to the south of the distribution at locality 17 (Churumuco).

### 3.2 Partitioning genomic variation

**FIGURE 5:**
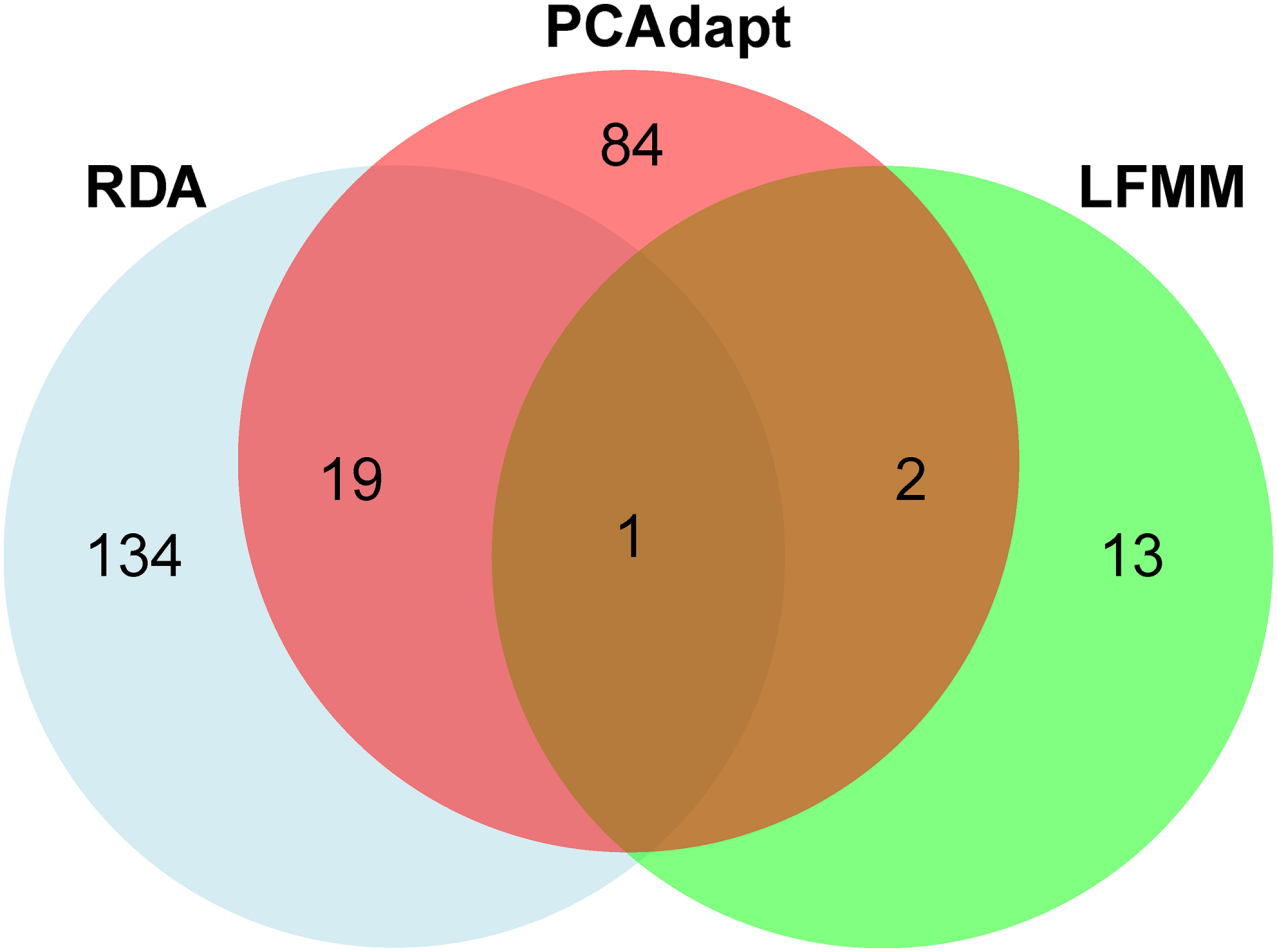
Numbers of significant outlier SNPs detected by three different methods for genome scans.

From the full set of SNPs (5311), 253 (4.76%) outlier SNPs were detected using the three detection methods. RDA was the least conservative (154 SNPs), PCAdapt was intermediate (106 SNPs), and LFMM was the most conservative (16 SNPs). According to the RDA, 75 SNPs were correlated to temperature seasonality (bio4), 21 SNPs to annual precipitation (bio12) and precipitation of warmest quarter (bio18), 16 SNPs to precipitation of coldest quarter (bio19), 14 SNPs to precipitation of driest quarter (bio17), and 9 SNPs to annual mean temperature (bio1). According to LFMM analysis, bio18 was the variable with the most correlated SNPs (9), followed by bio1 (4 SNPs), and bio19 (3 SNPs). The three methods detected only one candidate SNP in common, and only 22 candidate SNPs were shared across two methods (Figure 5).

Using neutral and putatively adaptive SNPs, we also carried out variation partitioning analysis to determine the relative contributions of environmental, geographic, and wind connectivity factors to the genomic variation. For the total data set, the results of the RDA model, including all parameters, showed a significant effect of these three factors on the variation of the 5289 neutral SNPs (adj. *R* ^2^ = .063, *p* < .001; Table 2). The analysis of individual factors revealed that the environmental variables explained 1.8% (adjusted *R* ^2^ = .018, *p* < .001; Table 2) and the spatial autocorrelation captured by MEMs explained 1.9% (adjusted *R* ^2^ = .019, *p* < .001; Table 2). In contrast, the wind‐only model was not significant.

**TABLE 2:** Redundancy analysis (RDA) to partition genetic variation in *Ficus pringlei* into environmental factors (env), spatial autocorrelation (space), wind connectivity (wind), and their combined effects.

	Entire dataset^a^		North lineage^b^		South lineage^c^	
	Neutral	Putative adaptive	Neutral	Putative adaptive	Neutral	Putative adaptive
Combined fractions						
F ~ env	0.041***	0.292***	0.021***	0.132**	0.024***	0.118***
F ~ space	0.041***	0.254***	0.008**	0.096^ns^	0.025***	0.126***
F ~ wind	0.020***	0.167***	0.003*	0.039^ns^	0.007**	0.043**
Individual fractions						
F ~ env|space+wind	0.018***	0.113***	0.018**	0.080**	0.001^ns^	0.037**
F ~ space|env + wind	0.019***	0.074***	0.006^ns^	0.000^ns^	0.008^ns^	0.034*
F ~ wind|space+env	0.001^ns^	0.013**	0.003^ns^	0.000^ns^	0.002^ns^	0.001^ns^
F ~ env + space|wind	0.006	0.020	0.002	0.007	0.018	0.048
F ~ space+wind|env	0.001	0.000	0.000	0.034	0.001	0.010
F ~ wind+env|space	0.002	0.000	0.001	0.015	0.006	0.000
F ~ env + space+wind	0.063***	0.373***	0.030***	0.087**	0.035***	0.162***
Total confounded^d^	0.015	0.166	0.000	0.000	0.000	0.034
Total unexplained	0.937	0.627	0.970	0.912	0.965	0.838

*Note*: The analysis was conducted for both neutral (5289 SNPs) and putative adaptive (22 SNPs) data across the entire dataset and for each lineage individually. Calculated with the adjusted *R* ^2^ value, any negative values were treated as null in this calculation. F: Dependent matrix of allele frequencies per individual. ***p < .001; **p < .01; *p < .05; ^ns^not significant. Significance of confounded fractions between factors was not tested.

^a^ Neutral: 5 Moran eigenvector maps (MEM) and 6 env variables; Putative adaptive: 3 MEM, 6 env variables.

^b^ Neutral: 3 MEM and 4 env variables; Putative adaptive: 3 MEM, 2 env variables.

^c^ Neutral: 3 MEM and 4 env variables; Putative adaptive: 2 MEM, 1 env variables.

^d^ Total confounded = Total individual fractions cofounded between various combinations of env, space, and wind.

In the case of SNPs identified as putatively adaptive, the model including all parameters explained significant proportions of the genomic variation (adjusted *R* ^2^ = .373, *p* < .001; Table 2). Pure environmental variables explained 11% of the outlier SNP variation (*R* ^2^ adj. = .113, *p* < .001; Table 2), spatial autocorrelation explained 7% (*R* ^2^ adj. = .074, *p* < .001; Table 2), and wind contributed to 1.3% (*R* ^2^ adj. = .013, *p* < .001; Table 2). For both datasets, 1.5–16% of the explained variation was confounded between the effects of spatial autocorrelation, environmental, and wind (‘Total confounded’ in Table 2). In summary, the population divergence of *F*. *pringlei* was strongly determined by the joint effect of environmental, geographical, and wind factors, however, environmental variables were more important than geography and wind in the putatively adaptive dataset.

**FIGURE 6:**
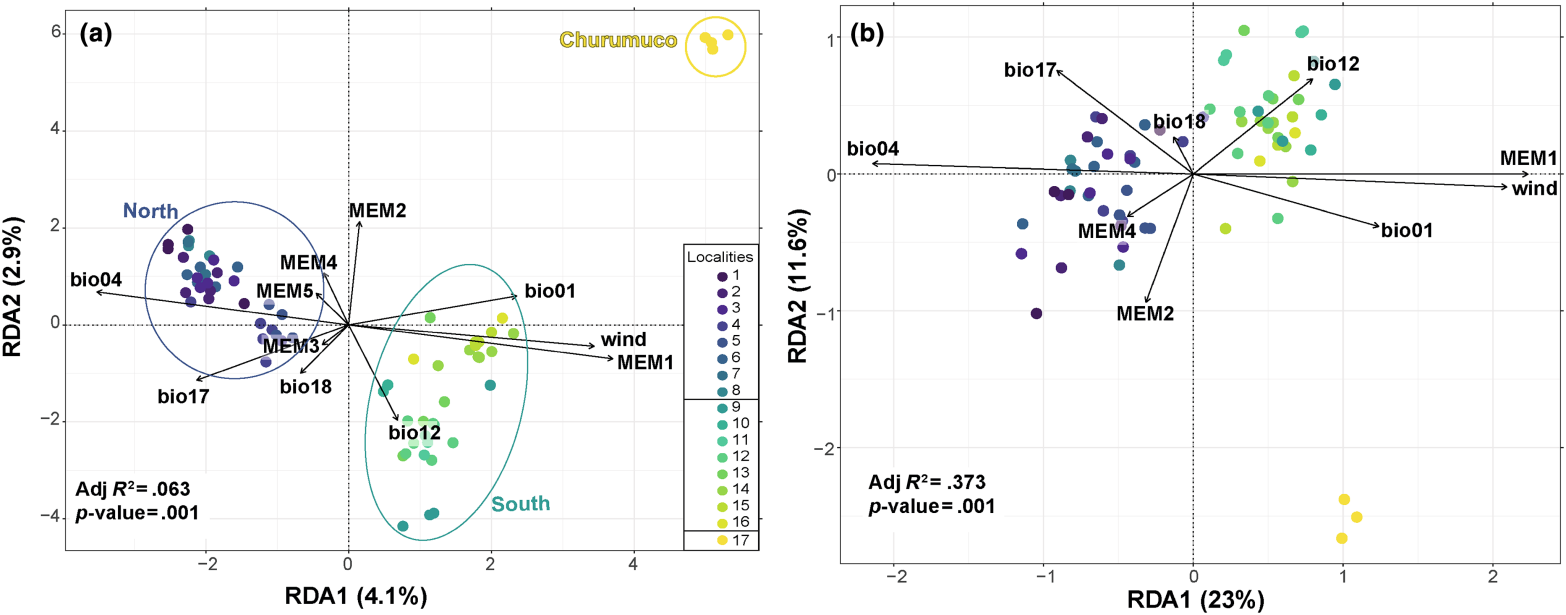
Distance‐based redundancy analysis biplot reveals the influence of environmental, geographic, and wind connectivity factors on the genomic variation in *Ficus pringlei*. (a) Biplot of simple RDA with the all‐neutral dataset. (b) Biplot of simple RDA with all putative adaptive dataset. Arrows represent environmental variables that drive the observed population structure. Moran's eigenvector map (MEM) vectors represent geographical isolation at different spatial scales. Dots represent individuals. bio1: annual mean temperature; bio4: temperature seasonality; bio12: annual precipitation; bio17: precipitation of driest quarter; bio18: precipitation of warmest quarter; wind: wind connectivity.

**FIGURE A2:**
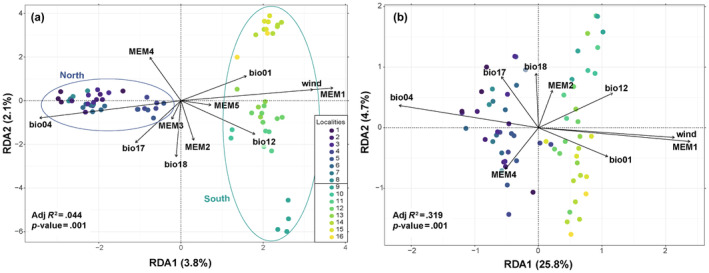
Distance‐based redundancy analysis biplot reveals the influence of environmental, geographic, and wind connectivity factors on the genomic variation in *Ficus pringlei* without Churumuco locality. (a) Biplot of simple RDA with the all‐neutral dataset. (b) Biplot of simple RDA with all putative adaptive dataset. Arrows represent environmental variables that drive the observed population structure. Moran's eigenvector map (MEM) vectors represent geographical isolation at different spatial scales. Dots represent individuals. bio1: annual mean temperature; bio4: temperature seasonality; bio12: annual precipitation; bio17: precipitation of driest quarter; bio18: precipitation of warmest quarter; wind: wind connectivity.

Simple RDA biplots (Figure 6) with putative adaptive and neutral datasets showcased a population structure consistent with the three genomic lineages identified by PCA. RDA biplots also provided insights into the relative importance of environmental gradients. For example, individuals of the north were associated with greater temperature seasonality and lower mean annual temperature. In contrast, individuals from the south and Churumuco were associated with higher mean annual temperature and lower temperature seasonality (Figure 6 and Figure A2 in Appendix). On the second RDA axis, individuals from Churumuco were negatively associated with precipitation‐related variables. Wind dispersal patterns are represented by one‐way travel time (hours) for the area where *F. pringlei* is located. Black dots indicate individuals sampled for this study. Warmer colors represent shorter travel times, indicating greater connectivity, while colder colors indicate less connected areas. Temperature seasonality (bio4) emerged as the most crucial environmental variable in both datasets. This variable showed only a high correlation with MEM1 (*r* = −.94) and wind connectivity (*r* = −.90), underlining its interconnected role in influencing genetic variation.

The findings in the northern and southern lineages are like those observed in the overall data set. The model combining environmental, spatial variables, and wind explained significant proportions of the genomic variation in both neutral (north: adj. *R* ^2^ = .030, *p* < .001; south: adj. *R* ^2^ = .035, *p* < .001; Table 2) and in the putatively adaptive dataset (north: adj. *R* ^2^ = .087, *p* < .001; south: adj. *R* ^2^ = .162, *p* < .001; Table 2). Examination of individual variables revealed that environmental factors emerged as the main determinant explaining genetic variation in both the neutral and potentially adaptive data from the north, as well as in the putatively adaptive data from the south (Table 2). However, a significant portion of the variation is unexplained by the evaluated factors.

## 4 DISCUSSION

Despite their low population density, several tropical plants present moderate or even high levels of genetic diversity and, in many cases, an excess of heterozygotes (Eguiarte et al., 1992; Heer et al., 2015; Nazareno & Carvalho, 2009; Teixeira & Nazareno, 2021). In the case of *F*. *pringlei*, the moderate levels of *H*_e_ and negative values of *F*_IS_ for all locations may be related to its perennial life form, cross‐pollination, pollen dispersal over long distances, and random mating of individuals facilitated by the frequent reproductive asynchrony (Rojas‐Cortés et al., 2022), as has been suggested for other *Ficus* species (Heer et al., 2015; Nazareno et al., 2013; Nazareno & Carvalho, 2009).

A perennial habit can allow greater production of seeds and pollen in multiple reproductive seasons, which would increase the genetic variability of the population (Hamrick & Godt, 1996). Cross‐pollination and the dispersal of pollen over long distances also could allow a greater exchange of alleles between individuals and populations (Hamrick et al., 1992; Hamrick & Godt, 1996), as observed in individuals with lower relatedness values. On the other hand, excess heterozygosity in species with small populations and highly seasonal environments, such as *F*. *pringlei*, may result from variability in the timing of flowering and seed maturation. This variability could increase the probability of mating with individuals carrying different alleles, facilitate random mating, and ultimately increase heterozygosity (Gates & Nason, 2012). However, in dioecious species of the genus *Ficus*, an excess of homozygotes is frequently observed (e.g., Chen et al., 2008; Dev et al., 2011; Wang et al., 2009). Therefore, it would be important to further investigate how asynchrony between and within trees, as well as mating patterns in species with different reproductive systems, contribute to the observed heterozygosity.

### 4.1 Genomic variation

Topographic complexity, including geographic barriers, and environmental heterogeneity have been identified as influential factors driving genetic differentiation in some *Ficus* species (Deng et al., 2020; Harrison, 2005; Heer et al., 2015; Honorio Coronado et al., 2014; Rodriguez et al., 2017; Souto‐Vilarós et al., 2019). Our RDA analysis for *F*. *pringlei* supports the notion that the arrangement of mountain complexes, along with environmental factors and prevailing wind direction, may contribute to genetic differentiation, potentially leading to the emergence of new ecotypes. Despite these influences, genetic differentiation in this microendemic species remains relatively low. Specifically, environmental variables emerged as a better predictor of genomic variation, suggesting a signal of local adaptation. However, a significant portion of genomic variation was also associated with spatial autocorrelation, suggesting that neutral processes, in conjunction with environmental interactions, may play a role in shaping genetic diversity.

Identifying SNPs associated with temperature and precipitation seasonality by GEA methods, together with genomic variation driven primarily by environmental variables at putatively adaptive SNPs, suggests that *F*. *pringlei* has adapted to specific environmental conditions. Therefore, temperature and precipitation may potentially guide this local adaptation in *F*. *pringlei*, as well as in other plants (Li et al., 2019; Linhart & Grant, 1996). Temperature variations may lead to the selection of distinct ecotypes in the northern and southern regions of the TMVB, while precipitation, especially in the south, may favor different ecotypes from east to west.

At the phenotypic level, the adaptive process may manifest in plant phenology and interactions with pollinators. For example, in the northern regions of the TMVB, marked by higher temperature seasonality, various developmental stages of syconia can be found within the same *F*. *pringlei* tree, as has already been recorded (Rojas‐Cortés et al., 2022). This dynamic could contribute to maintaining wasp populations in seasonal environments (Gates & Nason, 2012; Smith & Bronstein, 1996), as well as to the isolation of populations and their subsequent differentiation over time (Martin et al., 2009).

On the other hand, south of the TMVB, specifically in Churumuco, the interplay of high temperatures and low precipitation can pose a significant challenge to the longevity of wasps. Despite their inherent mobility, these wasps, which typically live for a brief period (1–2 days) outside the syconium, are notably susceptible to abiotic stress (Gigante et al., 2021; Jevanandam et al., 2013; van Kolfschoten et al., 2022; Xu et al., 2021). Consequently, the environmental conditions in this region might diminish the lifespan of wasps and impact their dispersal capacity (Gigante et al., 2021; Xu et al., 2021). This potential reduction in wasp longevity could be reflected in the high relatedness values observed among *F*. *pringlei* individuals, along with limited genetic connectivity with other localities.

Patterns of gene flow revealed by FEEMS could be attributable to geography, particularly the uplands to the west of TMVB and Sierra Madre del Sur, which had been previously identified by analyzing gene flow patterns for several species found on both sides of the TMVB (Anguiano‐Constante et al., 2021; Gándara & Sosa, 2014; López‐Barrera et al., 2021; Ruiz‐Sanchez & Ornelas, 2014). However, there is low differentiation between individuals north and south of the TMVB in *F*. *pringlei* that may be generated by a somewhat limited gene flow, which may be possible only throughout the lower elevation zones located to the east of the TMVB, as has been suggested for other dry habitat species (Contreras‐Negrete et al., 2021). Although levels of differentiation are not high, evidence suggests that geography plays an important role in the genetic structuring of *F*. *pringlei*, even with the potential for high gene flow facilitated by small wasps that move passively with the wind (Ahmed et al., 2009; Nason et al., 1998).

Wasp dispersal is a critical factor in host genetic variation, as wasp movement directs pollen transport and gene flow (Liu et al., 2015; Nazareno et al., 2013). Wind, through gusts and atmospheric currents, plays a fundamental role in directing wasps to new areas, thus facilitating genetic mixing between host populations (Ahmed et al., 2009). At the regional level, wind patterns explain only a modest portion of the putatively adaptive genetic variation in *F*. *pringlei*, suggesting a direct influence on genetic diversity related to local adaptations. However, when examining individual lineages, there is no significant effect of wind patterns. While the proportion of adaptive genetic variation explained by wind may be relatively modest, this factor holds significance in influencing the local adaptation of *F*. *pringlei* through the guidance of pollinator dispersal. In contrast, neutral variation seems to be less affected by the influence of wind.

The neutral genetic variation total of *F*. *pringlei* appears to be susceptible to the influence of environmental conditions, spatial autocorrelation, and even the interplay between both factors. Demographic history and gene flow processes might have been molded by distinct variations in their immediate environment, as has been suggested in other plants (Liu et al., 2023). Additionally, both neutral and adaptive data indicated the existence of induced spatial dependence, suggesting that geographical proximity may align with large‐scale environmental similarities (Borcard et al., 2011). However, when examining neutral genetic variation between lineages, none of the evaluated factors offers an explanatory response in the south. In contrast, the north lineage exhibits an explanatory correlation solely with the environment, highlighting the intricate complexity of these factors.

Finally, substantial variation remains unexplained, which is common in genetic studies using these methods (Gibson & Moyle, 2020; Liu et al., 2023). This could be attributed, in part, to the population structure that may not be fully explained by spatial variables of the MEM, potentially due to geographic heterogeneity present in the Mexican Transition Zone. It is essential to remember that RDA, by limiting itself to modeling linear associations between explanatory variables and SNPs, may overlook nonlinear statistical relationships (Gibson & Moyle, 2020). Furthermore, other unmeasured forces could also be acting, like the localized seed dispersal that in some species may be more restricted than pollen movement (Honorio Coronado et al., 2014; Souto‐Vilarós et al., 2019; Yu & Nason, 2013). The main seed dispersers of *F*. *pringlei* are still unknown, but Rojas‐Cortés et al. (2022) suggested that birds may be dispersal vectors since the mature syconia are red. The size and behaviors of frugivores affect seed‐mediated gene flow. In the future, to better understand the drivers of the genetic structure of *F*. *pringlei*, our results should be combined with information on the dispersers and their contribution to gene flow.

### 4.2 Local adaptation and speciation dynamics

Local adaptation plays a crucial role in the genetic structuring of the mutualistic relationship between *Ficus* and fig wasps and is potentially responsible for species‐level divergence and the process of co‐speciation (Rodriguez et al., 2017; Souto‐Vilarós et al., 2019; Tian et al., 2015). Restricted gene flow by climatic conditions and by non‐random mating in wasps would facilitate their isolation and genetic differentiation, which, in turn, would limit the gene flow of their hosts and eventually lead to parapatric speciation in both interactants of the mutualism (Orsini et al., 2013; Souto‐Vilarós et al., 2019; Tian et al., 2015). This local adaptation and speciation process probably occurs initially in wasps since they have shorter generation times, which would explain why the one‐to‐one relationship between the host and pollinating wasps frequently breaks (Darwell et al., 2014; Rodriguez et al., 2017; Souto‐Vilarós et al., 2019; Yu et al., 2019). This process has been mainly observed in dioecious species, probably due to its grouped establishment and lower pollen dispersal in comparison to monoecious figs (Nazareno et al., 2013). However, the local adaptation process may occur in monoecious species in regions with highly heterogeneous environments, such as where *F*. *pringlei* is found.

## 5 CONCLUSIONS

This study provides information on the diversity and the mechanisms underlying the genomic variation of *F*. *pringlei*. Although a weak or negligible genetic structure is often assumed for the *Ficus* species, in *F*. *pringlei* we detected three distinct genomic lineages, suggesting limited gene flow influenced mainly by climatic conditions. Populations of *F*. *pringlei*, north and south of TMVB and Sierra Madre del Sur, exhibit minimal genetic differentiation, suggesting that this mountain range may not act as an impassable barrier to gene flow. Its porous nature allows for some degree of gene exchange between populations.

Our analysis emphasizes the role of wind in shaping the genetic diversity of *F*. *pringlei*. Although not the main factor, it also plays a crucial role in structuring putatively adaptive genetic variation, highlighting the intricate complexity of the various factors influencing genetic variation in the species. Therefore, in this microendemic species, the arrangement of mountain complexes, along with environmental factors and predominant wind direction, may contribute to genetic differentiation and the emergence of new ecotypes. However, despite these influences, genetic differentiation in this species remains relatively low.

Further studies are warranted on the reproductive phenology of *F*. *pringlei* and the role of seed dispersers in gene flow to enhance our understanding of this complex interaction. Additionally, comprehensive studies on the genetic variation of wasps and other relevant aspects would be valuable to gain a more integrated understanding of the diversification of this species and the process of cospeciation.

## AUTHOR CONTRIBUTIONS

**Ángela P. Rojas‐Cortés:** Conceptualization (lead); data curation (lead); formal analysis (lead); funding acquisition (supporting); investigation (lead); methodology (equal); project administration (equal); visualization (lead); writing – original draft (lead); writing – review and editing (equal). **Jaime Gasca‐Pineda:** Conceptualization (supporting); data curation (supporting); formal analysis (supporting); investigation (supporting); methodology (equal); writing – review and editing (equal). **Antonio González‐Rodríguez:** Conceptualization (supporting); resources (equal); writing – review and editing (equal). **Guillermo Ibarra‐Manríquez:** Conceptualization (supporting); funding acquisition (lead); investigation (equal); project administration (equal); resources (equal); writing – review and editing (equal).

## FUNDING INFORMATION

This work was supported by the Dirección General del Personal Académico (DGAPA)‐Programa de apoyo de investigación e Innovación Tecnológica (PAPIIT IN211620) of the Universidad Nacional Autónoma de México (UNAM).

## CONFLICT OF INTEREST STATEMENT

All authors claim no conflict of interest.

## Data Availability

Genotypes and descriptions of individual and environmental data are archived on DRYAD https://datadryad.org/stash/share/4FOC8McXwWntboOnwbjT4RTxiXQtqMFn3dnH9N1aJ6A.
